# Etiological aspects of solitary bone cysts: comments regarding the presence of the disease in two brothers. Is the genetic theory sustainable or is it pure coincidence? – Case report

**Published:** 2015

**Authors:** A Miu

**Affiliations:** * Department of Pediatric Orthopedics, “M.S. Curie” Hospital, Bucharest, Romania

**Keywords:** solitary bone cyst, siblings, genetic disease, humerus, TEN

## Abstract

Beginning the study of benign tumors of the bone in children and adolescents, a group of diseases that have in common the clinical aspects, evolution, and surgical treatment, genetic theory in the etiology of the solitary bone cyst, can be sustained by some cases of siblings with the same disease.

This paper presents the particular case of two brothers, treated in our clinic for the same condition: solitary bone cyst of the proximal humerus. The two brothers were admitted with the same symptoms, the localization was the same. Because of the genetic studies regarding this condition, we think that it is an interesting aspect of this pathology. This study also tried to find the most appropriate approach in the treatment of these tumors.

## Definition

Solitary bone cyst is a benign bone lesion, consisting of one single cavity, lined by a membrane, containing serous yellowish liquid/ or tainted with blood, found in the proximal metaphysis in children and adolescents. It may appear in any bone, but it is typically found in either the proximal humerus, or the proximal femur. A solitary bone cyst often leads to the thinning of the adjacent areas of bone, so that fracture or pain from microfracture may occur. When the cysts are immediately adjacent to a growth plate, they are referred to as active cysts, and when they have achieved some distance from the growth plate, they are considered latent cysts. The active cysts have a bad outcome, disturbing the growth and deforming the area (coxa vara/ valga). These lesions are rarely found in adults, thereby a spontaneous resolution may be possible [**1**,**2**,**5**,**9**,**12**].

**Epidemiology**

Frequency - it occurs mostly in children between 5 and 15 years old, with a peak at 9 years old.

- a solitary bone cyst affects males twice as often as females

- these tumors constitute approximately 3% of all bone tumors, occupying the third place, after ostheochondroma and fibrous dysplasia [**5**,**12**].

**Etiology**

The specific etiology of the solitary/ unicameral bone cyst has not been elucidated. Many theories have been proposed; none of them was demonstrated.

a) the haemoragic theory, studied by Pommers, assumes that an intramedullary haemorrage leads to blood encapsulation, lined by a fibrous membrane. The rise of the blood pressure of the lesion leads to the erosion of the adjacent tissues. Consequence: the thinning of the bone cortex.

b) Mickulicz presents another theory, in which a hiatus may appear in the long bones, after a trauma. This theory is sustained by several cases in which solitary bone cysts appeared after major trauma of the bones.

c) Jaffe & Lichtenstein demonstrated an ossification failure in the bone metaphysis appeared in the fast growing process [**9**].

d) Johnson & Kindred highlighted the dysplastic theory, suggested by the fact that the membrane is formed by mesenchymal elements.

e) Cohen proposed the vascular theory. According to this theory, the principal etiologic factor is the blockage of the drainage of interstitial fluid in a rapidly growing and rapidly remodeling area of the growing bone. Chigira and a group of Japanese researchers studied the pressure of the cystic liquid, and found that it was higher compared to the contralateral normal bone (2-3mmHg higher) [**6**-**8**].

f) the genetic theory (the subject of this paper) is reported by a group of Brazilian researchers (Vayego & colab), who have found genetic abnormalities in a pediatric patient with a solitary bone cyst in the right distal femur. These genetic disorders consist of a complex aberration of chromosomes 4, 6, 8, 12, 16, 21. Further studies of the same patient revealed specific mutations associated with the amino acid substitution (arginine for tryptophan, arginine for serine). Molecular and genetic studies helped elucidate the mechanisms involved in the etiology and outcome of the benign and malignant lesions of the bones. These studies have proven useful in the potentially malignant diseases, also in the relapses of the solitary bone cysts, despite the treatment. In these particular cases, the relapse percent was between 35 and 70. Richkind et al. reported a simple translocation involving the short arm of chromosome 16 and the long arm of chromosome 20, in a curetted specimen of a 9-year-old boy with solitary bone cyst [**13**,**15**].

The aim of the study was to discuss the possibility of the genetic transmission of the solitary bone cyst.

## Material and method

- The case of two brothers was brought to discussion, both presented with solitary bone cyst of the proximal humerus. Both were treated in the “M.S. Curie” Pediatric Orthopedic Clinic, Bucharest, during 2007 and 2013. Pure coincidence or genetic predisposition deal that the lesion is located at the same level.

- It is the only case of this kind in the clinic statistics in the past 5 years.

- only one case of this kind was found in the literature (a case of two monozygotic twins with SBC of the proximal humerus [**16**]).

- the retrospective study of children treated with Solitary bone cyst, during the 5 years period, took into consideration only the surgical cases. From 2007 to 2013, 118 patients were admitted in our clinic with this diagnostic.

**Fig. 1:**
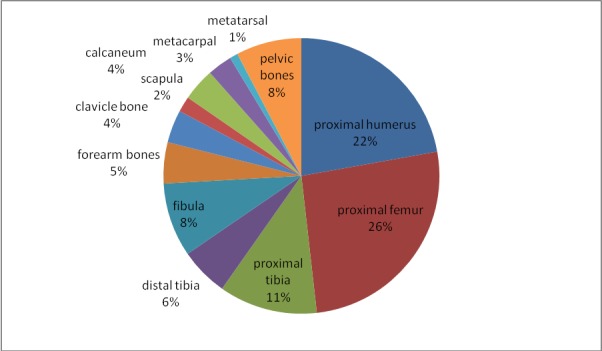
SBC distribution

**Case I (2010)**

- a 5-year-old boy, presented in the E.R. with right shoulder pain, after a minor trauma. The emergency X-ray showed a pathological fracture of the proximal right humerus due to a solitary bone cyst, with minimal displacement. The initial treatment was immobilization for 30 days in a cast. The conservative treatment was justified by the age of the patient and the slight displacement of the fracture. At the 4 months follow-up, the cystic lesion persisted and the fracture was healed. The decision to pursue surgical intervention in patients with solitary bone cysts was individualized. Due to our experience, the cyst resolution after conservative treatment resulted in 20% of the cases. Thereby, it was decided to perform a surgical intervention in this particular case.

Also, the cyst index was used as a predictor factor of the future risk of a fracture, defined by A. Kaelin & D. MacEwen as the area of the cyst divided by the diameter of the diaphysis of the same bone. In this case, the C. I. was measured as 4 [**10**,**11**].

**Fig. 2:**
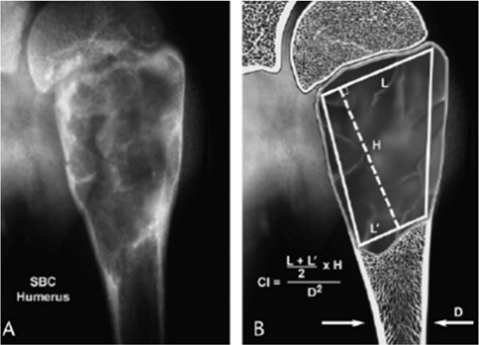
Cyst index according to Kaelin

A surgical intervention consisting of a minimum invasive biopsy and osteosynthesis with TEN was performed [**14**].

Surgical technique:

-under fluoroscopic imaging, the proper entry site of the nails was selected

-a small skin incision was made, 2 cm above the condyle area, by spreading the muscles we reached the bone surface. After drilling a hole (with an awl), 2 elastic nails, manually prebent, were introduced, according to the retrograde technique, under fluoroscopic control.

-another small incision was made above the cystic area (C-Arm imaging); after reaching the bone cortex, a biopsy-resection, curettage of the cavity, cauterization of the cystic walls and breaking all of the cystic trabeculae, were made.

-a casting was not mandatory

The great advantage of the elastic nails is that they permit small movements of the fragments, and the healing is accelerated by callus distraction. Also, the technique is accessible, but the presence of a C-Arm is mandatory. The “stiffness” of the elastic nails is related to the human bone.

The nails were extracted after 12 months, radiologically, the healing was achieved. No sequel appeared in this patient [**3**,**4**,**14**].

**Fig. 3a:**
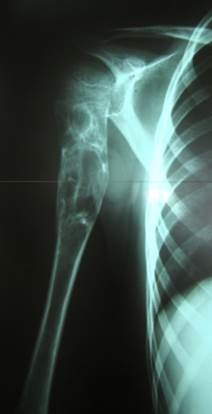
Initial aspect

**Fig. 3b:**
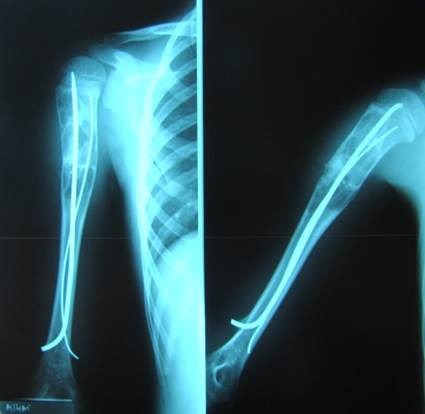
Postoperatory aspect

**Fig. 3c:**
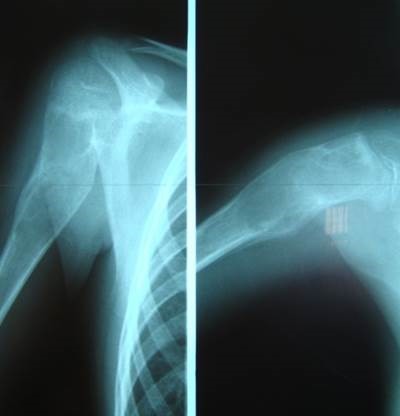
1 year after

**Case II (2012)**

An 11-year-old boy (brother of the previous patient) came to the E.R. with the same symptoms, which appeared after a minor trauma during a football game. X-Ray showed a pathological fracture of the proximal humerus (solitary bone cyst). Because of the patient’s age, in whom the indication of the elastic nails was the better choice, it was decided as treatment for the surgical intervention. The bone defect was also larger than in its brother’s case, so, the cavity was filled with Pro-Dense, as a supplementary measure. 

The radiological follow-up (3, 6 months) showed perfect healing. The nails will be extracted one year after surgery was performed.

**Fig. 4:**
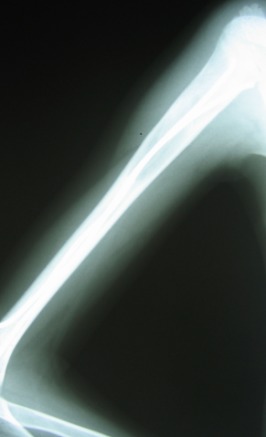
Six months follow-up

## Discussion

The genetic studies could determine the possibility of genetic transmission of some disorders, clarifying the controversy regarding their etiopathology. Seeing that this lesion was benign, the treatment was accessible and easy to perform, the outcome was predictable good, and the researchers had to focus on therapy than on etiology. Nevertheless, the uniqueness of this case brought to attention the matter of a genetic disorder. As a medical curiosity worth mentioning-the single case in the literature was mentioned by Takahiro G, Tetsuo N et al. They studied the case of two monozygotic twins, with mirror image SBC of the humerus. The two boys underwent surgery, histological findings of the curetted specimen supported the preoperative diagnosis of SBC [**16**].
